# *COPEWithME*: The Role of Parental Ability to Support and Promote Child Resilient Behaviors During the COVID-19 Emergency

**DOI:** 10.3389/fpsyg.2021.732745

**Published:** 2021-10-14

**Authors:** Isabella Lucia Chiara Mariani Wigley, Eleonora Mascheroni, Francesca Bulletti, Sabrina Bonichini

**Affiliations:** ^1^Department of Developmental and Social Psychology, University of Padua, Padua, Italy; ^2^0-3 Center for the at-Risk Infant, Scientific Institute, IRCCS Eugenio Medea, Bosisio Parini, Italy

**Keywords:** COVID-19, stress-related behavior, family well-being, parental resilience, child resilience

## Abstract

The COVID-19 pandemic has led to lockdown in many countries and Italy was the first one interested in Europe. The lockdown strategy is an essential step to curb the exponential rise of COVID-19 cases, but it is very demanding for the population involved and especially for children and their families. The aims of the present study are: (a) to explore the psychometric properties of the *COPEWithME* questionnaire, a new tool to evaluate parents' ability to support and promote child resilient behaviors, (b) to investigate the relation between parents' resilience and their ability to support and promote child resilient behaviors with child resilience and child stress-related behaviors assessed during the COVID-19 outbreak. Participants (*N* = 158 mothers, with 6- to 11-years-old children, 53% female), who were volunteers and anonymous, filled out an online questionnaire composed by CD-RISC 25, PMK-CYRM-R, and *COPEWithME*. With regard to the *COPEWithME*, validation exploratory factor analyses revealed a one-factor solution of 18 items. The *COPEWithME* positively correlates both with mothers' resilience and with children's resilience. Mediation analysis showed that the association between mothers' resilience and children's stress-related behaviors was mediated by the mothers' ability to support and promote child resilient behaviors. The *COPEWithME*, to our knowledge, is the first measure of parents' ability to support and promote resilient behaviors in school-age children, a key parenting skill that may help children in dealing with stressful situations such as the COVID-19 outbreak. These findings represent useful insights to advance mental health interventions in the post-pandemic phases suggesting focusing on a family's resources and resilience processes.

## Introduction

The novel coronavirus (COVID-19) rapidly spread all over the word, affecting many countries severely. Italy was the first European country affected by the COVID-19 pandemic outbreak. To ensure infection control and prevent disease transmission, lockdown measures have been implemented, such as quarantine, social-distancing, school closure, and suspension of non-essential activities, requiring in many cases remote working and families' adjustment to 24/7 interaction. Despite these strategies having proven to be effective in containing the spread of the virus, several studies reported that the COVID-19 outbreak has generated a considerable amount of stress among the population (Xie et al., 2020), resulting in high psychological costs and several negative outcomes for children and their families (Liu et al., 2020). As a result, the risk for developing negative behavioral and psychological outcomes in the developmental age is real and warrants attention. Besides, it is important to consider that addressing risk factors alone loses sight of those protective factors, such as resilience, that are essential to advancing science, services, education, and policies aimed at understanding how children and adolescents respond to crises and how they can be supported (Dvorsky et al., 2020). We refer to resilience as a process, built through learning and memory mechanisms (DiCorcia and Tronick, 2011; Lee et al., 2016), which support the individual and promote well-being when exposed to high levels of stress or adversity (Ungar and Theron, 2020). In fact, recent studies in this regard are suggesting that resilience is not a fixed trait, but instead it can be learned and improved (Booth and Neill, 2017). Due to global lockdown and social distancing, the nuclear family appears to be the main place of learning potential functional coping and adaptation strategies in the context of the COVID-19 outbreak. In this view, the current work would like to better understand how parents' resilience and their ability to teach resilient behaviors to children can influence both child resilience and stress-related behaviors assessed during the COVID-19 outbreak (Prime et al., 2020).

### Families Dealing With Pandemic

#### Children

Different evidence suggested that the COVID-19 emergency negatively affected children's physical and psychological well-being (Jiao et al., 2020; Liu et al., 2020). School closure and changing in children's daily routine might interfere both with healthy habits such as outdoor activity, daylight exposure, and with psychological adjustment due to the social distancing measures that prevented in-person relationships with peers for a long time (Cluver et al., 2020). Studies conducted during previous pandemics (i.e., A-H1N1) reported that quarantine and social distancing measures were associated with an increase of 30% of post-traumatic stress disorder rates and psychological distress in children (Sprang and Silman, 2013). As far as the current pandemic emergency concerns, data collected in the COVID-19 affected areas in China revealed that children aged 3 to 18 displayed high degrees of clinginess, inattention, and irritability. It was observed that preschool children displayed fear of losing their caregivers, while school-aged children manifested higher levels of inattention (Jiao et al., 2020). Similarly, a study conducted on a sample of 3,245 Italian children and adolescents revealed that behavioral problems were present in 65% of preschool children and in 71% of school-aged children. Specifically, children under the age of 6 years displayed increased irritability, sleep disorders, and internalizing problems, and children over the age of 6 years showed higher levels of somatic complaints, sleep problems, emotional instability, and irritability (Uccella et al., 2021).

However, beyond addressing adverse outcomes, it is important to focus on protective factors as well as they can buffer the effects of adverse experiences exposure (Dvorsky et al., 2020). Individual resilience, for example, has been shown to preserve psychological well-being even after adverse and traumatic life events in children (Banyard et al., 2017). Resilience can be defined as an individual process that enables positive adaptation in the face of stressful situations and is generally regarded as a multidimensional concept that includes learning and memory processes (DiCorcia and Tronick, 2011; Lee et al., 2016). Several researches identified a wide range of protective and promoting factors associated with positive adjustment in response to adversities determined by both individual and external factors (Zolkoski and Bullock, 2012; Dvorsky et al., 2020). What is implied is that the ability to be resilient can be developed and enhanced from early childhood, throughout the lifespan. Due to measures to prevent the spread of COVID-19, the relationship with the primary caregivers became the core systems supporting the child's ability for self-regulation, learning, problem-solving, motivation to adapt, persistence, and hope, all crucial skills for the development of child resilient behaviors (Masten, 2011). Therefore, understanding the relation between parents' and children's resilience comes in the light of evidence showing that children's adjustment is largely contingent on the overall climate and relationships in a family (Browne et al., 2015).

#### Parents

The COVID-19 emergency has put a strain not only on children, but on families' systems in general disrupting habits and requiring a substantial reorganization of time and space, too (Prime et al., 2020). A recent survey conducted in the U.K. showed that parents were experiencing increased stress during the coronavirus outbreak, as they were trying to balance caring responsibilities, home schooling, and working from home (Power, 2020). Evidence coming from prior pandemics (i.e., Ebola) suggested that parents experience greater psychological distress compared to adults without children (Kamara et al., 2017). Moreover, parents are also at higher risk of burnout due to chronic rates of parental stress along with inadequate resources and support (Griffith, 2020). This is of particular concern because parents must manage children 24/7 because of social confinement and school closure. Notably, family factors such as parental distress and irritability have an impact on child outcomes by exacerbating negative and non-functional reactions (Mikolajczak et al., 2018). Prime et al. (2020) recently published a conceptual model illustrating the complex ways in which pandemic disruptions and stress will infiltrate and impede family functioning through negative effects on caregiver well-being and cascading, bidirectional effects on child adjustment (Prime et al., 2020). Their model also aligns with another relevant theoretical model of caregiver resilience by Gavidia-Payne et al. (2015), in which child and family characteristics impact family functioning which, in turn, affects caregiver well-being and self-efficacy, all contributing to quality and resilient caregiving. Moreover, recent research on Italian women revealed that mothers manifest higher symptoms of anxiety disorders compared to mothers without children during the COVID-19 lockdown (Benassi et al., 2020).

Besides, research highlights several caregivers' psychosocial competences that may assist families' and children's coping and adaptation strategies during an adverse situation (Dercon and Krishnan, 2009). Parental resilience is defined as parents' ability to deliver competent, quality parenting to children despite adverse circumstances (Gavidia-Payne et al., 2015). This process has been found to play a key role in families dealing with stressful situations. Researchers have found that children who have been exposed to different kinds of trauma (e.g., war trauma and natural disaster) tend to have a higher level of psychological well-being when the adults in their lives are available to soothe and help them with their overwhelmed emotions (Costa et al., 2009; Diab et al., 2019).

However, to the best of our knowledge, no study has previously inquired how maternal resilience can help children facing a stressor, such as the COVID-19 pandemic, as well as their ability to teach children resilient behaviors. To answer this question, we developed a specific tool to assess parental perception of teaching resilience to their children, namely, the *COPEWithME*.

### The *COPEWithMe* Questionnaire

The child's functional adaptation strategies and individual resilience can be promoted and supported by those systems that are very close to the child, such as relationships with competent and caring adults (i.e., parents, grandparents, teachers, and educators). Dynamic developmental system models of resilience in children highlight that child resilience can depend not only on the adaptive systems within the child, but also on the resilience of their family members (Masten and Cicchetti, 2016; Hostinar and Miller, 2019). During the lockdown period, the close family system became the primary venue for supporting coping and adaptation in the COVID-19 outbreak. In the pandemic scenario, the role of parents was further emphasized by the lack of children's contact with other adults (e.g., teachers and grandparents), assuming a unique key role in promoting and supporting child resilience and their children's positive adjustment (Doty et al., 2017; Masten and Motti-Stefanidi, 2020).

As during previous pandemics or other traumatic events such as a natural disaster (e.g., Hurricane Katrina), parents had to take care of all the children's needs, various organizations and structures have mobilized with the aim of providing psychological support to families. Beyond national and international organizations specialized in health issues (e.g., WHO, IMH), in times of crisis, other channels, such as blogs and social media, play a key role in supporting and helping families (Wiederhold, 2020). Due to the global lockdown and social distancing, these kinds of resources have proven to be essential, offering useful resources to cope with the virus outbreak (Saud et al., 2020). In this regard, several blogs (e.g., Pandemic Parenting, Info About Kids, Chelsea Lee Smith) gave parents specific advice on how they can represent a model of resilience implementing behavioral, emotional, and cognitive processes that could help their children in the enhancement of coping skills and abilities (child resilience). Specific tips included: make the child practice waiting patiently, give the child independence to try new activities, let the child deal with their emotions by not belittling their feelings, and not giving them everything they want.

However, to the best of our knowledge, no studies have investigated whether these strategies were valid, reliable, and effective in successfully supporting and promoting children's resilient behaviors during COVID-19. To this aim, the *COPEWithME* questionnaire was developed including as items key behaviors mostly suggested to parents from institutional guidelines and social tools to promote resilience in children. In the current work, after validating and examining the psychometric properties of this questionnaire, we explored possible associations between parents' and children's resilience, parents' ability to support and promote resilient behaviors in their school-aged children, and children's resilience and stress-related behaviors observed during lockdown due to the COVID-19 pandemic in Italy.

### Aims

The overall aim of the present study was to explore the role of parents' resilience and their ability to support and promote child resilient behaviors toward child resilience and child stress-related behaviors during the COVID-19 outbreak. To this end, three specific aims were outlined. First, starting from the literature on parents' and children's resilience and online sources of parenting advice (i.e., social tool), the questionnaire *COPEWithME* was developed and validated in order to assess parental perception of teaching resilient behaviors to their children. We expect to have a valid instrument to assess parental ability to teach resilient behaviors and to implement behavioral, emotional, and cognitive abilities that could enhance coping skills in their children. Second, the impact of the COVID-19 outbreak on children's well-being was evaluated and, in line with previous research, an increase in stress-related behaviors was expected. The last aim of the study was to test if parental resilience could influence children's stress-related behaviors through their ability to support and promote child resilient behaviors and child resilience. It was expected that greater parental resilience could be associated with higher ability of teaching it to the child and with better child individual skills and resources and, finally, to less stress-related behaviors assessed during the COVID-19 outbreak.

## Method

### Participants and Procedures

Data were collected immediately after the end of the first Italian lockdown between May 18 and June 4, 2020, using an online anonymous survey. All participants were parents of children aged between 6 and 11 years, recruited through snowball sampling. Parents, who were contacted using a mailing list, signed a consent to be informed via e-mail. Participants' inclusion criteria included: be more than 18 years old, be an Italian native speaker, and be a parent of a child between the ages of 6 and 11. They were asked to follow a link that led to the survey, and they were informed that pressing the link was deemed as consent to participate. The survey was completed by 166 parents (95.2% mothers) who experienced containment and restrictive measures due to the international health emergency (Prime Minister Decree, March 9, 2020; Government, 2020). The study protocol was approved by the Psychology Ethics Committee of the School of Psychology, University of Padua (number of protocol: D6B09283C9694D9C8EFCFBD33C713130).

### Measures

#### Parental Perception of Teaching Resilient Behaviors

The original version of the *COPEWithME* questionnaire administered in the online survey consisted of 24 items describing possible behaviors the parent taught to their child to be resilient (i.e., be able to deal with it on their own, when they have difficulties doing something, without a parent immediately rushing to help them). For each item, parents were asked to assign a score on a 5-point scale, where 0 means “not at all” and 4 corresponds to “very much.” In this scale, the higher the score, the greater the parent effort to teach resilience. The final version of the *COPEWithME* included 18 items.

#### Sample Demographics and COVID-19 Related Variables

The socio-demographic section included items asking general information (e.g., parent's age, civil status, educational level, who responded to the survey) and items regarding family composition and characteristics (e.g., children's age and gender). A specific section was devoted to information about the impact of containment on the household on their work organization.

#### Parental Resilience

In order to evaluate parental resilience, the Italian version of the Connor-Davidson Resilience Scale 25-Item Score (CD-RISC 25, Connor and Davidson, 2003) was used. The scale consists of 25 items rated on a 5-point Likert scale, ranging from 0 (*not at all agree*) to 4 (*totally agree*). The total score can range from 0 to 100 and the higher the score obtained, the greater the subject's resilience. Regarding the reliability in this study, Cronbach's α was measured indicating a very good internal consistency of the scale (α = 0.93).

#### Child's Individual Resources

For the purpose of the present study, to assess a child's individual resources, the Personal Resilience subscale of the Person Most Knowledgeable version of the Child and Youth Resilience Measure-Revised (PMK-CYRM-R) was used (Jefferies et al., 2018). This subscale of PMK-CYRM-R includes 10 items to be answered by the parents on a 5-point Likert scale, ranging from 0 (*not at all*) to 4 (*very much*), with a maximum total score equal to 50 points. A higher score is associated with a greater degree of perceived resilience. In this subscale, a child's individual resilience includes personal and social skills (such as ability of problem solving, cooperation, and awareness of personal strengths). The reliability measure of this subscale used for this study obtained a Cronbach's α of 0.76, indicating an acceptable internal consistency.

#### Child's Stress-Related Behaviors

In order to assess the impact of COVID-19 outbreak on child well-being, in terms of displaying a specific target behavior, an *ad hoc* list of eight stress-related behaviors was created. Stress-related behaviors included: (1) difficulty standing still; (2) concentration difficulties; (3) nervousness and irritability; (4) tendency to cry for no reason; (5) difficulty falling asleep; (6) restless sleep with awakenings; (7) food refusal; and (8) excessive food seeking. Parent was asked to indicate the presence of each behavior before (i.e., past stress-related behavior, which referred to the presence of the child's stress-related behavior before the COVID-19 outbreak) and during the confinement period (i.e., actual stress-related behavior, which referred to the presence of the child's stress-related behavior during the COVID-19 outbreak).

### Data Analysis Plan

Data were analyzed with the IBM SPSS 22 software and PROCESS macro (Hayes, 2012). Variables were first examined for the presence of outliers and tested for normal distribution of the items (kurtosis and asymmetry ranging from −1 to +1). Exploratory factor analysis (EFA) was run in order to test the factor structure of the *COPEWithME*. A maximum likelihood exploratory factor analysis with a Promax rotation was performed for factor extraction. *COPEWithME* internal consistency was then evaluated through McDonald's ω. Regarding children's stress-related behaviors, paired sample *t*-tests were performed to analyze possible changes between children's past stress-related behavior and actual stress-related behavior. Once stress-related behaviors that significantly increased during the confinement experience were identified, another maximum likelihood exploratory factor analysis with a Promax rotation was performed to identify possible overall factors related to a child's stress-related behaviors. Preliminary correlations were performed in order to test associations between the included variables (parents' and children's resilience, *COPEWithME* score, children's stress-related behaviors). Finally, a sequential mediation model was tested, including parents' resilience as a predictor, *COPEWithME* score, and children's resilience to cope with stressful situations as a mediator, with children's stress-related behaviors as an outcome. The model was controlled for child's age.

## Results

### Descriptive Statistics

Data were collected from 166 families whose socio-demographic characteristics are summarized in Table 1.

**Table 1:** Participants' socio-demographic characteristics.

**MOTHERS' CHARACTERISTICS (*****N*** **= 158)**		
**Age (range)**	***M* = 43.27 years**	***SD* = 4.20**
	**N**	**%**
	***F* = 158 *M* = 8**	**92.54.8**
**Marital Status (** ***N*** **= 158)**		
** Married/Cohabitant**	**150**	**94.9**
** Divorced/Separated**	**7**	**4.4**
** Single**	**1**	**0.6**
**Education (*****N*** **= 158)**		
** Middle school**	**16**	**45.6**
** High school**	**72**	**25.3**
** Bachelor degree**	**40**	**10.1**
** PhD/Master**	**30**	**19**
**CHILDREN'S CHARACTERISTICS (*****N*** **= 158)**		
**Age (range 6–11 years)**	***M* = 8.88 *years***	***SD* = 1.41**
	**N**	**%**
**Gender (*****N*** **= 154)**		
** Male**	**72**	**48.1**
** Female**	**82**	**51.9**
**Parents' workplace during COVID-19 (*****N*** **= 153)**		
	**N**	**%**
Workplace	50	31.6
Home (smart-working)	50	31.6
Workplace and home	25	15.8
Unemployed	28	17.7

Descriptive statistics regarding parental resilience, *COPEWithME* scores, PMK-CYRM-R, and children's stress-related behaviors (pre- and during the COVID-19 outbreak) are summarized in Table 2.

**Table 2:** Descriptive statistics of the included variables.

	**M**		**SD**	
CD-RISC 25	63.78		16.86	
*COPEWithMe*	2.599		10.47	
PMK-CYRM-R	22.56		0.59	
**Child stress-related behaviors**	**Past stress-related behavior**		**Actual stress-related behavior**	
	**M**	**SD**	**M**	**SD**
Factor 1				
Difficulty standing still	1.53	0.63	1.82	0.73
Concentration difficulties	1.65	0.58	2.07	0.69
Nervousness and irritability	1.61	0.54	1.99	0.68
Tendency to cry for no reason	1.26	0.45	1.54	0.72
Food refusal	1.13	0.33	1.23	0.47
Excessive food seeking	1.20	0.45	1.39	0.66
Factor 2				
Difficulty falling asleep	1.27	0.52	1.72	0.77
Restless sleep with awakenings	1.22	0.47	1.38	0.58

### *COPEWithME* Factor Structure: Exploratory Factor Analysis (EFA) and Reliability

A maximum likelihood exploratory factor analysis with a Promax rotation was conducted on the 24 items of the original version of the *COPEWithME*, in order to explore the factor structure and to examine the quality of the items in our sample. An initial analysis was run to obtain eigenvalues for each component in the data. Six components had eigenvalues over Kaiser's criterion of 1 and in combination explained 61% of the variance. However, this method is often criticized for retaining too many factors (O'Connor, 2000; Hayton et al., 2004), so we used Horn (1965) parallel analysis (PA) and Cattell (1966) scree method to determine the number of components. The PA revealed to extract only one factor. Moreover, the scree plot shows a clear inflection after component 1 that further justifies retaining only one component. The maximum likelihood exploratory factor analysis with a Promax rotation was re-run specifying a one-factor solution. Six items were drop out from the original version of the questionnaire. Items were dropped out considering two criteria: (1) item's communalities < 0.25; (2) factor loadings < |0.30| (Barbaranelli and D'Olimpio, 2007). Table 3 shows the final one-factor version of the *COPEWithME*, with the included items (*N* = 16) and their factor loadings. Bartlett's test of sphericity showed that Chi-square was significant at the < 0.001 level (χ^2^= 1224.021, *df* = 153), and the Kaiser-Mayer-Olkin measure of sampling adequacy was 0.87. This one-factor solution explained 35% of the variance. McDonald's ω for the final version of the questionnaire was 0.902, demonstrating very good internal consistency (Andrew and Jacob, 2020). To investigate the role of parents' ability to support and promote children's resilient behaviors during the COVID-19 outbreak, we computed the overall mean score of the 18 included items and considered this value in subsequent analyses.

**Table 3:** *COPEWithMe* items (Italian and English version) and EFA factor loadings.

	**Italian version**	**English translation**	**Item reliabilities**	**Factor loadings**
**Item**				
1	Ho insegnato a mio figlio/a ad: Aspettare il proprio turno (al ristorante o alle giostre) anche senza intrattenimento (tablet, videogiochi, cibo…)	I taught my son/daughter to:Wait his turn (at restaurants or rides) even without entertainment (tablets, video games, food.)	0.407	0.522
2	Prendere buone decisioni che avranno un effetto a lungo termine, anche se non sono semplici	Make good decisions that will have a long-term effect, even if they are not simple	0.419	0.580
3	Essere consapevole che le cose che possiede non soddisfano il desiderio di felicità	Be aware that the things he owns do not satisfy his desire for happiness	0.434	0.537
4	Affrontare le difficoltà e gli ostacoli	Deal with difficulties and obstacles	0.581	0.681
5	Avere un atteggiamento positivo verso gli impegni e i compiti scolastici	Have a positive attitude toward school commitments and assignments	0.653	0.741
6	Essere paziente quando gioca con gli altri bambini (o fratelli) soprattutto quando lo disturbano nei suoi giochi	Be patient when playing with other children (or siblings), especially when they disturb him in his games	0.453	0.540
7	Autocontrollarsi con gli strumenti elettronici (ne limita l'uso solo per momenti prestabiliti)	Self-monitor the use of electronic tools (limits their use only for predetermined moments)	0.481	0.529
8	Affrontare le differenti condizioni climatiche vestendosi adeguatamente	Cope with different weather conditions by dressing appropriately	0.395	0.455
9	Quando ha difficoltà nel fare qualcosa, riuscire ad affrontarlo da solo, senza che un genitore accorra subito in suo aiuto	When he has difficulties doing something, be able to deal with it on his own, without a parent immediately rushing to help	0.480	0.690
10	Non interrompere gli altri quando parlane e sa aspettare il suo turno	Not to interrupt others when speaking and can wait his turn	0.484	0.632
11	Essere esposto/a a nuove esperienze e riuscire bene al di fuori degli ambienti a lui/lei familiari	Be exposed to new experiences and doing well outside familiar environments	0.468	0.532
12	Quando deve trovare qualcosa, cercarlo da solo	When he needs to find something, look for it himself.	0.528	0.511
13	Prendersi cura dei suoi abiti (li rimette a posto, non li lascia in giro)	Take care of her/his clothes (puts them back, doesn't leave them lying around)	610	0.656
14	Fare del suo meglio a scuola, anche se richiede dei sacrifici	Do her/his best in school, even if it requires sacrifice	0.504	0.617
15	Essere cosciente delle sue responsabilità e doveri (es. rifarsi il letto, prendersi cura della propria igiene)	Be aware of his responsibilities and duties (e.g., making his own bed, taking care of his own hygiene)	0.615	0.653
16	Riuscire a trarre il meglio da ogni situazione ed è grato/a per quello che ha	Make the best of every situation and is grateful for what she/he has.	0.617	0.751
17	Vivere i propri sentimenti, soprattutto quelli negativi, non sminuendoli e aiutandolo ad affrontarli	Experience her/his feelings, especially the negative ones, not belittling them and helping her/him to deal with them	0.342	0.429
18	Ad aiutare gli altri	To help other people	0.313	0.405

### Child's Stress-Related Behaviors

Paired *t*-tests pointed out that from before to during the COVID-19 outbreak, all the eight behaviors assessed were significantly increased in children (Table 4).

**Table 4:** Comparison between child stress-related behaviors before and during COVID-19 outbreak.

**Stress-related behaviors changes in children from before to during COVID-19 outbreak**	***t***	***p***	***Cohen's d***
Difficulty standing still	−6.21	< 0.001	0.42
Concentration difficulties	−8.07	< 0.001	0.66
Nervousness and irritability	−7.63	< 0.001	0.63
Tendency to cry for no reason	−5.60	< 0.001	0.47
Difficulty falling asleep	−7.28	< 0.001	0.69
Restless sleep with awakenings	−4.01	< 0.001	0.31
Food refusal	−3.68	< 0.001	0.27
Excessive food seeking	−4.19	< 0.001	0.33

Due to the significant increase of all considered behaviors, we considered stress-related behaviors during the confinement period for subsequent analyses.

A maximum likelihood exploratory factor analysis with a Promax rotation was performed to identify possible child's stress-related behaviors (during the confinement) overall factors. Two different factors emerged. The first factor (factor 1) included six stress-related behaviors which referred to children's behavioral problems; the second factor (factor 2) included two stress-related behaviors which referred to sleep problems. Cronbach's α for factor 1 was 0.73, while for factor 2 was 0.70.

The mean scores of these two factors were computed. These two scores were used for the subsequent analyses. The overall mean score for factor 1 was 1.54 (*SD* = 0.59); the overall mean score for factor 2 was 1.67 (*SD* = 0.43).

### Preliminary Correlations

Regarding the relations between parental and child resilience, no significant correlation emerged between CD-RISC 25 and PMK-CYRM-R Personal Resilience subscale (*r* = 0.145, *p* = *0.070)*. Parental resilience was positively correlated also with the *COPEWithME* score: the more resilient a parent is, the better he or she teaches effective strategies to cope with stressful situations to his or her child (*r* = 0.311, *p*<*0.001)*. Finally, parental ability to teach effective strategies to cope significantly correlates with better child resilience to cope with COVID-19 (*r* = 0.562, *p*<*0.001)*. Regarding children's stress-related behaviors observed by parents during the confinement, a higher mean score of factor 1 (i.e., higher level of stress-related behavior) was significantly related to (a) poorer parental resilience (*r* = −0.213, *p* = *0.007*), (b) poorer parental ability to support and promote resilient behaviors (*r* = −0.319, *p*<*0.001)*, and poorer child resilience (*r* = −0.249, *p* = *0.002)*. No significant correlation emerged with the mean score of factor 2.

### Sequential Mediation Model

**Figure 1:**
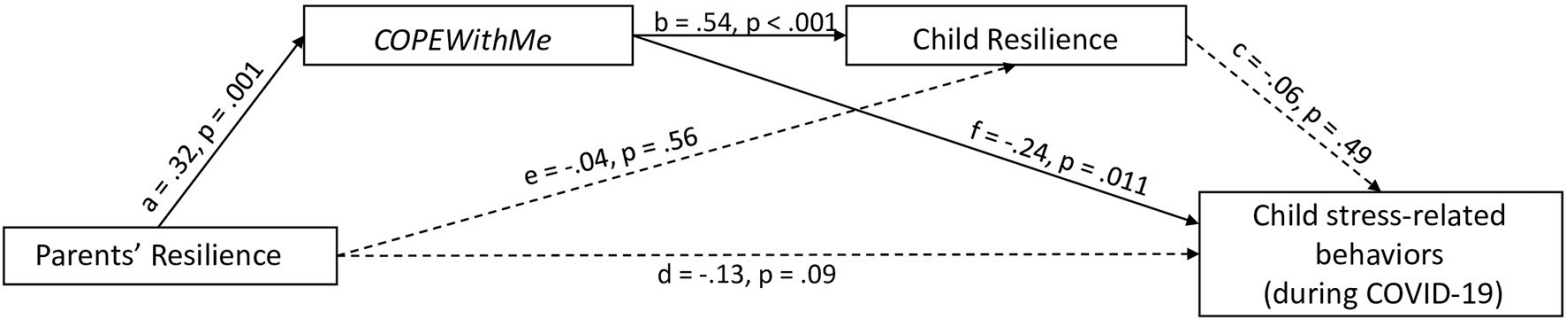
Sequential mediation model. The figure excludes the covariate of child age; however, it is included in tests of the model. Standardized effect coefficients are reported.

The model included parents' resilience as a predictor, *COPEWithME* score as first mediator, child resilience as second mediator, and child stress-related behaviors assessed during the COVID-19 outbreak as outcome (Figure 1). The model was controlled for the child's age. As a result, besides the significant positive effect of parental resilience on *COPEWithME* scores (*b* = 0.011, *s.e*. = 0.003, *p* < 0.001) and the one of *COPEWithME* scores on child resilience (*b* = 3.428, *s.e*. = 0.452, *p* < 0.001), we found a significant negative effect of parental ability to support and promote child resilient behaviors and child stress-related behaviors assessed during the COVID-19 outbreak (*b* = −0.178, *s.e*. = 0.069, *p* = 0.011). As parents' ability to support and promote child resilient behaviors decreased, child stress-related behaviors related to the COVID-19 pandemic increased. Overall, the three predictors explain the 16% of the variance observed in child stress-related behaviors assessed during the virus outbreak (*F*_(4,149)_ = 7.24, *p* < 0.001). Moreover, the indirect effect of parental resilience on child stress-related behaviors through *COPEWithME* scores is also significant (*b* = −0.002, *boostrap s.e*. = 0.001, *boostrap 95% C.I*.: −0.0048: −0.0003). Parents' ability to support and promote child resilient behaviors to deal with stressful situations emerged as a crucial factor in mediating the relation between parents' resilience and children's stress-related responses to the COVID-19 outbreak. The direct effect was not significant (*b* = −0.003, *boostrap s.e*. = 0.002, *boostrap 95% C.I*.: −0.0073: 0.0006).

## Discussion

The present study was designed at exploring the role of parents' resilience and their ability to support and promote child resilient behaviors in explaining child resilience and stress-related behaviors assessed during the COVID-19 outbreak. In our view, there are two main innovative aspects introduced by this work. First is the implementation of *COPEWithME* as the first tool to assess parents' ability to support and promote resilient behaviors in school age-children. Second, to the best of our knowledge, this study is one of the first to report the link between parental and children resilience in the first month after the end of quarantine in May 2020 in Italy.

The COVID-19 pandemic represents one of the most stressful recent events worldwide and poses a major challenge for the social, economic, and, above all, the psychological resources of the population, so it is very important to investigate the psychological impact of this event on families and children. In this regard, one relevant finding of the present study outlined that during the pandemic, different child stress-related behaviors significantly increased compared to before the COVID-19 outbreak. These findings are in line with several previous research studies emphasizing the dramatic effects of this pandemic in children (Gassman-Pines et al., 2020; Spinelli et al., 2020; Wang et al., 2020). It is therefore clear that children's psychological well-being must be at the heart of the post-pandemic recovery plan. Besides, apart from considering the negative effects of COVID-19, it is also important to take into account those factors that might act, at least partially, as protective factors, such as resilience and the ability to teach resilient behaviors to the most frail.

In order to assess parental perception of having taught effectively resilient strategies to their children, the *COPEWithME* questionnaire was first developed and then validated in the present study. Results showed a valid single factor structure of the *COPEWithME* final version and a strong correlation with the PMK-CYRM-R Individual Resources subscale that measures child capacity to be resilient, supporting a significative concurrent validity.

The central aim of the present research was to investigate the effects of parent resilience on child stress-related behaviors assessed during the COVID-19 pandemic, through the parents' ability to support and promote resilient behaviors and child resilience. As expected, greater parental individual resilience was associated with higher ability of teaching it to the child and with better children's skills and resources. These results are in line with previous findings (Dercon and Krishnan, 2009) that observed that parental personal competences and resources have a positive influence on children's personal coping strategies during an adverse situation. Notably, the consistency of these findings might indicate that this process is relatively independent from the type of stressful situation.

Moreover, contrary to our expectation, parental resilience does not significantly correlate and, consequently, does not directly predict child resilience. Our results seem to indicate that a child's ability to be resilient is supported by good parental function, rather than parental resilience alone. The ability of parents to support and promote resilience positively influences children's individual resources and positive adjustment. Children that live with parents who can be models of resilience promoting behavioral, emotional, and cognitive processes can also positively adapt in the face of stressful situations.

Another unexpected result emerged from the sequential mediation model tested. It showed that the association between parent resilience and child stress-related behaviors was mediated only by the parents' ability to support and promote resilient behaviors and not even by child resilience. As a result, a significant indirect pathway linking parent resilience, *COPEWithME* scores, and child stress-related behaviors emerged showing that the more resilient the parent, the better his or her ability to teach resilient behaviors to the child and the fewer difficulties the child experienced during the pandemic. It is important to point out that resilience does not necessarily suggest immunity against situational stressors. Rather, it can be considered as the ability to process and overcome an ongoing distress (Isokääntä et al., 2019). It is possible to identify two phases that can characterize resilience: adversity (i.e., the exposure to distress in potential trauma) and positive adaptation (i.e., resilience process). Notably, the resilience process is an ongoing interaction between one's personal strengths and weaknesses, and other significant factors in the daily environment (Ungar, 2015). In this framework one can infer that since children are less fully developed socially than adults and have no context in which to process events, they require more support both to promote their resilience and to prevent behavioral problems.

Several studies documented that resilience can buffer the negative consequences of stressful life events (Luthar et al., 2000), also supporting the view that increases in parental resilience could improve adaptive behaviors in children (Masten, 2011; Doty et al., 2017). In addition, confirming this previous evidence, our results expanded these findings suggesting that parental abilities to promote and support child resilient behaviors play a key role in children's positive adjustment in facing highly stressful events. As no direct effect of parent resilience on child stress-related behaviors emerged, the present findings suggest that being a resilient parent is not in itself a protective factor for the child. The significant effect emerges through the parent's ability to support and promote child resilient behaviors.

Overall, the present findings represent useful insights thinking about family interventions. For clinicians working with parents in the post-pandemic phase, it may be useful to focus on increasing the parents' ability to support child resilient behaviors, in order to achieve two substantial positive effects. First, effectively teaching the child to be more resilient makes the child able to functionally manage an adverse situation. Second, this makes the child more peaceful and, in turn, lowers the burden on the parent. Alongside the current need to think about support interventions among mental health-care providers for families and communities, there is also the need to understand which factors to focus on most. The results of the present study may represent useful insight to advance mental health interventions focusing on families' resilience processes.

Finally, our study has some strengths and limitations. To the best of our knowledge, it is one of the first studies to provide an opportunity to investigate the relation between parents' and children's resilience and parental perception of promoting resilient behaviors in their children, conducted in the month after a lockdown was imposed by the Italian government. However, our study has some limitations. First, the study used a cross-sectional design for evaluating how parents' resilience and their ability to teach resilient behaviors to children can influence both child resilience and stress-related behaviors assessed during the COVID-19 outbreak. Cross-sectional designs are a pragmatic approach that help to constrain time and costs while at the same time identifying key variables and potential relations, as for example, the relations between ability to teach resilient behaviors and child adjustment. In order to enhance our findings, a longitudinal investigation will be essential for stronger causal inferences. Second, a further limitation of the present study was the limited sample size. The current report provides initial investigation of the *CopeWithMe* in Italian mothers. To strengthen our results, further investigations in broader samples are needed. Third, the convenience sampling method may prevent the generalization of our findings. Moreover, since due to the lack of a father's response we only included the mother's response in the final sample, one should be careful about interpreting the present results, since there could be possible parental role/gender differences in terms of promoting and supporting child resilient behavior. Future research using *CopeWithM*e is needed to investigate the role played by fathers in promoting and supporting child resilient behavior during a stressful/traumatic situation. In addition to these aspects, the temporal window of data collection must be considered as a limit, as it started after the end of quarantine and lasted one month. We must be aware that these data are just a snapshot of a contingent situation. We, of course, do not have data about participants' resilience before the quarantine. Finally, since this was not the focus of the current study, only a few main psychometric properties of *CopeWithM*e were tested in the current work. However, there would be some important aspects that future research should consider. First, researchers increasingly suggest that the Classical Test Theory (CTT) approaches such as exploratory factor analysis cannot provide a complete representation of the psychometric properties of an instrument. CTT is not congruent with the idea that resilience is not a fixed trait but instead it can be learned and improved (Booth and Neill, 2017). In order to consider resilience as a state (Ye et al., 2020), further analyses should include different analytical approaches in agreement with the Generalizability Theory. Also, our results suggested a one-facto solution for the *CopeWithM*e; thus, a future important next step would be to confirm that result with model tests for the unidimensional test theoretical models. Moreover, for practical reasons, here we decided to compute the overall mean score of the 18 included items. However, future studies are required to verify whether considering the mean values, as we did, is justified.

## Conclusions

In emergency situations, such as that caused by the COVID-19 pandemic, identifying effective ways to reduce stress and increase resilience has become a mandate for people from all walks of life, ages, professions, and socioeconomic backgrounds. In particular, families and children are among the first to be focused on and schools and other institutions around them must continue to develop a public health framework to understand the various risks and protective factors of COVID-19 and its aftermath. Several studies highlighted that the ability of a system to cope with an atypical stress situation improves the ability of co-occurring systems (Twum-Antwi et al., 2020). Therefore, as the *COPEWithME* resulted in a valuable tool to collect data about parental ability to support and promote child resilient behaviors, it could be useful in planning supporting interventions. Specifically, the *COPEWithME* could be used to plan intervention for caregivers (e.g., teachers, parents) aimed at improving child individual resources to cope with a stress. Thus, present results highlight the importance of thinking about interventions designed to improve child well-being by supporting the parents.

## Data Availability Statement

The raw data supporting the conclusions of this article will be made available by the authors, without undue reservation.

## Ethics Statement

The studies involving human participants were reviewed and approved by Psychology Ethics Committee of the School of Psychology, University of Padua (number of protocol: D6B09283C9694D9C8EFCFBD33C713130). The patients/participants provided their written informed consent to participate in this study.

## Author Contributions

IM and EM contributed to data analysis. IM, EM, and SB contributed to interpretation of findings, drafting, and final approval of manuscript. FB contributed to data acquisition. All authors contributed to the article and approved the submitted version.

## Conflict of Interest

The authors declare that the research was conducted in the absence of any commercial or financial relationships that could be construed as a potential conflict of interest.

## Publisher's Note

All claims expressed in this article are solely those of the authors and do not necessarily represent those of their affiliated organizations, or those of the publisher, the editors and the reviewers. Any product that may be evaluated in this article, or claim that may be made by its manufacturer, is not guaranteed or endorsed by the publisher.
