# Supplementary material for: COPEWithME: The Role of Parental Ability to Support and Promote Child Resilient Behaviors During the COVID-19 Emergency
Source: Front Psychol. 2021 Oct 14;12:732745. doi: 10.3389/fpsyg.2021.732745 (PMC8552018; doi:10.3389/fpsyg.2021.732745)
Supplement: Supplementary file 1 [file Data_Sheet_1.docx]

Table S1. Parallel Analysis output for the *COPEWithME*

| Root | Raw Data  Eigenvalues | Means Random Data  Eigenvalues | Percentile Random Data  Eigenvalues |
| --- | --- | --- | --- |
| 1.00 | 7.598149 | 1.787041 | 1.918600 |
| 2.00 | 1.615116 | 1.645624 | 1.724967 |
| 3.00 | 1.455637 | 1.549733 | 1.621596 |
| 4.00 | 1.356634 | 1.465440 | 1.531778 |
| 5.00 | 1.292188 | 1.391226 | 1.444542 |
| 6.00 | 1.224494 | 1.318516 | 1.381634 |
| 7.00 | .985095 | 1.251717 | 1.313127 |
| 8.00 | .936187 | 1.193606 | 1.242522 |
| 9.00 | .840743 | 1.138745 | 1.191562 |
| 10.00 | .799771 | 1.080974 | 1.119820 |
| 11.00 | .696636 | 1.031846 | 1.069851 |
| 12.00 | .671803 | .982419 | 1.022624 |
| 13.00 | .598367 | .932387 | .972280 |
| 14.00 | .598367 | .932387 | .972280 |
| 15.00 | .529124 | .836689 | .876770 |
| 16.00 | .491744 | .792741 | .834129 |
| 17.00 | .398098 | .748641 | .791411 |
| 18.00 | .376193 | .702467 | .743167 |
| 19.00 | .349373 | .658077 | .702803 |
| 20.00 | .318981 | .616990 | .660022 |
| 21.00 | .294004 | .570303 | .621086 |
| 22.00 | .209517 | .477579 | .532685 |
| 23.00 | .209517 | .477579 | .532685 |
| 24.00 | .190441 | .416038 | .465431 |

Fig. S1. Scree plot depicting Eigenvalue against component number


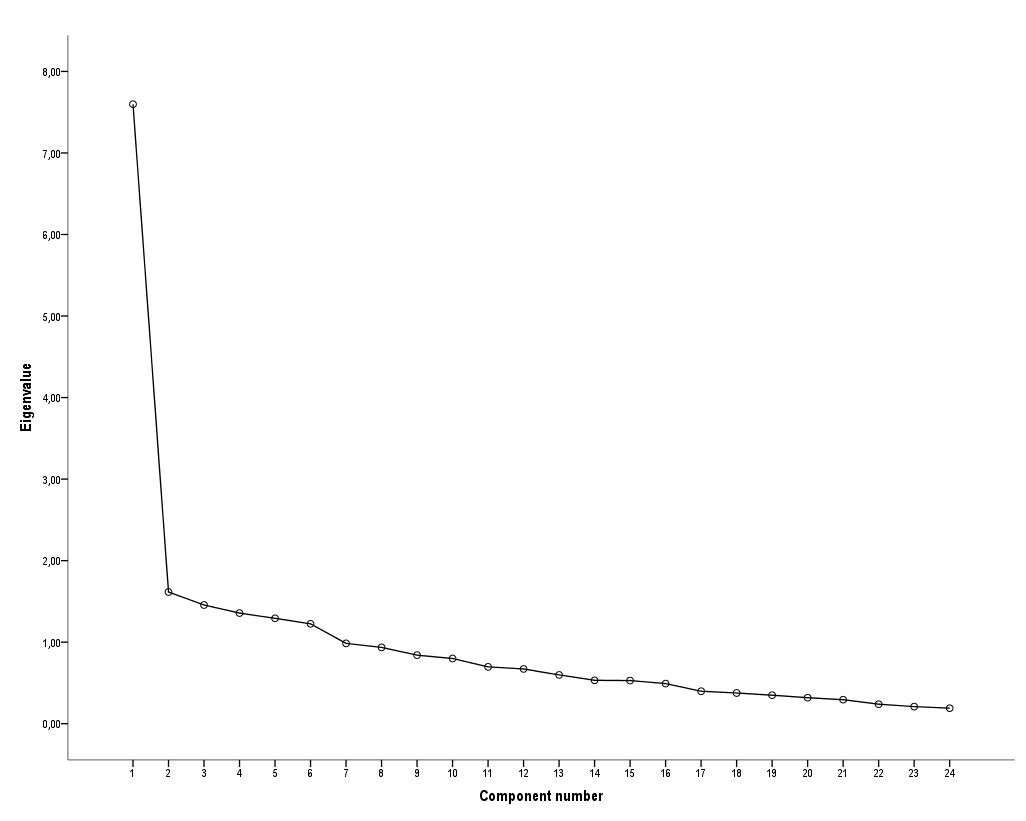


Table S2. Standardized indirect effects, ratio of indirect to total effect and ratio of indirect to direct effect of X (Parents’ Resilience) on Y (Child stress-related behaviors during COVID-19).

|  | Completely standardized indirect effect of X on Y | | | |
| --- | --- | --- | --- | --- |
|  | Effect | Boot SE | Boot LLCI | Boot ULCI |
| Total | **-.086 *** | .043 | -.189 | -.020 |
| Ind 1 | **-.078 *** | .045 | -.189 | -.010 |
| Ind 2 | .003 | .016 | -.006 | .033 |
| Ind 3 | -.011 | .008 | -.049 | .019 |
|  | Ratio of indirect to total effect of X on Y | | | |
|  | Effect | Boot SE | Boot LLCI | Boot ULCI |
| Total | .386 | .379 | .072 | 1.271 |
| Ind 1 | .349 | .364 | .040 | 1.261 |
| Ind 2 | -.012 | .044 | -.167 | .031 |
| Ind 3 | .049 | .108 | -.105 | .285 |
|  | Ratio of indirect to direct effect of X on Y | | | |
|  | Effect | Boot SE | Boot LLCI | Boot ULCI |
| Total | .628 | 10.467 | -3.049 | 18.131 |
| Ind 1 | .568 | 10.983 | -2.209 | 15.067 |
| Ind 2 | -.019 | .354 | -1.318 | .072 |
| Ind 3 | .079 | 1.472 | -.417 | 1.821 |

Note: Ind 1: Parents’ Resilience -> *COPEWithMe* -> Child stress-related behaviors (during COVID-19); Ind 2: Parents’ Resilience -> Child Resilience -> Child stress-related behaviors (during COVID-19); Ind 3: Parents’ Resilience -> *COPEWithMe* -> Child Resilience -> Child stress-related behaviors (during COVID-19); Boot SE: the mean of the indirect effect estimates calculated across all bootstrap samples; Boot LLCI: the lower limit of the 95% confidence intervals for population value of the indirect effects; Boot ULCI: the upper limit of the 95% confidence intervals for population value of the indirect effects; Bold values indicate statistical significance (p <0.05); * 95% Confidence intervals remains strictly positive or negative and do not cross zero.
